# The Effect of Thermocycling on the Translucency and Color Stability of Modified Glass Ceramic and Multilayer Zirconia Materials

**DOI:** 10.7759/cureus.6968

**Published:** 2020-02-12

**Authors:** Ghadeer Aljanobi, Zeyad H Al-Sowygh

**Affiliations:** 1 Department of Prosthetic Dental Sciences, College of Dentistry, King Saud University / Ministry of Health, Riyadh, SAU; 2 Department of Prosthetic Dental Sciences, College of Dentistry, King Saud University, Riyadh, SAU

**Keywords:** high translucent zirconia, zirconia, zirconia-reinforced lithium silicate, translucency, color stability, thermocycling

## Abstract

Aim

The aim of this in vitro study was to evaluate the new, multilayered, translucent zirconia and enhanced glass-ceramics to determine if their translucency (TP) and color stability (ΔE) are affected by thermocycling at 10,000, 30,000, and 50,000 cycles.

Materials & methods

Two pre-shaded, multilayer zirconia products: Prettau^®^ 2 Dispersive^® ^(PRT) and Prettau^®^ 4 Anterior^®^ Multi^® ^(PRTA), and two glass-ceramic: IPS e.max CAD^®^ HT (E.max) and Vita Suprinity^®^ HT (VS) were used. All were prepared and sectioned to get plate specimens with dimensions 12.5x14.5x1 mm (n=12) for each material. The L*a*b* values were recorded using a spectrophotometer before and after thermocycling for 10,000, 30,000, and 50000 cycles. The translucencies of the specimens were calculated using the TP formula and the color changes were giving by the color differences ΔE formula at each interval.

Results

One-way analysis of variance (ANOVA) was used to analyze the data followed by Scheffe’s post-hoc test and multiple paired t-tests (P < 0.05). There was a statistically significant higher TP for E.max before (16.2) and after aging (16.9) (p<0.001**). All the tested groups showed a statistically significant increase in their TP at different intervals. PRT showed significantly higher ΔE (p<0.001**) after 50,000 cycles.

Conclusion

There was a mild but significant increase in translucency in both Zirconia and glass-ceramic after thermocycling. In addition, all materials showed a significant color change with time, however, this is not clinically perceptible.

## Introduction

Esthetic and physical durability are two of the prime factors that clinicians consider when selecting dental ceramic materials [1]. As translucency is one of the most important factors in matching the natural appearance of teeth, lithium disilicate is well-proven for its high esthetic apparent. However, its major limitation is the low fracture strength, which has led to the development of a series of ceramic materials with high crystalline content to withstand the masticatory forces and mechanical stresses.

Zirconia-based dental ceramics are stronger and tougher materials than glass-ceramics [2-3]. Monolithic yttria-stabilized tetragonal zirconia polycrystal (Y-TZP) was introduced to overcome the fracture and chipping of the veneering porcelain from the opaque substructure zirconia material [4-5]. However, it is still showing lower translucency than glass-ceramics. To overcome that limitation, translucent Y-TZP was made commercially available by several brands. There are several factors that influence the translucency of Y-TZP and can be broadly divided into extrinsic and intrinsic factors. The manufacturer is responsible for the intrinsic factors and can include the crystal content and the size of the crystals, increasing the yttria, introducing lanthanum oxide (La_2_O_3_) in the composition, and lowering the alumina content and the sintering temperature [6-9]. The extrinsic factor is usually in the processing by the laboratory technician during the staining procedure. Manufacturers of newer zirconia systems claim that the materials have improved translucent properties.

There is also an attempt to enhance the strength of lithium disilicate by adding 10% of zirconium dioxide (ZrO_2_) in its composition, which believed to reinforce the lithium disilicate by inhibiting the crack propagation. This led to the introduction of a new subcategory called zirconia-reinforced lithium silicate with promising mechanical and esthetic properties [10].

Recently, a new polychromatic and pre-colored highly translucent zirconia material was produced by a few companies. The blocks come smooth, with natural color transitions. It disperses the color evenly within the block as a result of its special manufacturing process. According to the manufacturer, this material can be used in the anterior areas due to its excellent aesthetic properties and it was proposed to replace lithium disilicate [11]. Possessing significant translucency results is important to accept the claims of enhanced esthetics, made by manufacturers of these new types of dental ceramics.

The aims of this in vitro study were twofold: to evaluate the translucency of the new multilayer zirconia and zirconia-reinforced lithium silicate in comparison with lithium disilicate at a thickness of 1 mm and to evaluate the effect of thermocycling at 10,000, 30,000, and 50,000 cycles on their translucency and color stability. The first null hypothesis stated that there would be no significant differences in the translucency of multilayer zirconia and zirconia-reinforced lithium silicate as compared to lithium disilicate at a thickness of 1 mm. The second null hypothesis stated that there would be no significant differences in translucency and the color change of the tested materials after aging by thermocycling.

The clinical selection of ceramic systems is based on the mechanical and optical properties of materials. With the introduction of new esthetic material options in the market, the choice made by the clinician pertains to the case at hand and the individual weightage given to esthetics and strength. The rationale behind this study is to shed light on the optimum choice of ceramic system and crown material to be used for the esthetic area.

## Materials and methods

Four ceramic materials for monolithic dental crowns were selected for this study with a total of 48 samples (n=12). The materials selected were lithium disilicate (IPS e.max CAD®) (E.max), which served as the control group and three test materials, namely, multilayer posterior zirconia (Prettau® 2 Dispersive®) (PRT), multilayer high translucent zirconia (Prettau® 4 Anterior® Dispersive®) (PRTA), and zirconia-reinforced lithium silicate (Vita Suprinity®) (VS). Each was in the form of commercially available computer-aided design (CAD)/computer-aided manufacturing (CAM) blocks with a shade A2. High translucent (HT) blocks were used for both VS and E.max (Table 1).

**Table 1:** Materials grouping

	Material	Basic chemical structure	Manufacturer
Zirconia	Prettau^®^ 2 Dispersive^®^ (PRT)	unknown	Zirkonzahn GmbH, Bruneck, Italy
Highly translucent zirconia	Prettau^®^ 4 Anterior^®^ Dispersive^®^ (PRTA)	unknown	Zirkonzahn GmbH, Bruneck, Italy
Zirconia-reinforced lithium silicate ceramic	Vita Suprinity, (VS)	SiO_2_ (56-64%), ZrO_2_ (8-12%), Li_2_O (15-21%), La_2_O_3_ (0.1) and Pigments (< 10%)	Vita Zahnfabrick, Bad Säckingen, Germany
lithium disilicate ceramic	IPS e.max CAD (E.max)	SiO_2 _(57–80%), Li_2_O (11-19%), K_2_O (<13%), P_2_O_5_ (<11%), ZrO_2 _(<8%), ZnO (<8%), Al_2_O_3_<5%, MgO(<5%) and coloring oxides	Ivoclar Vivadent, Schaan, Lichtenstein

**Figure 1:**
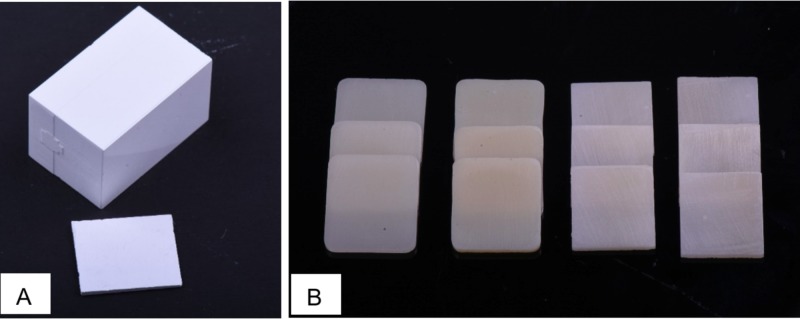
A: Milled zirconia block before slicing and sintering; B: The prepared specimens after crystallization and sintering

The specimens’ dimensions were set at 14.5 mm (length L) × 12.5 mm (width W) × 1 mm (height H). For both PRT and PRTA, the cutting dimension was determined by accounting for the 20% shrinkage that occurred during sintering. Blocks with dimensions 18.2 x13.9 mm were milled from pre-sintered zirconia using a CAD/CAM milling machine (M5 Heavy Metal Milling Unit, Zirkonzahn, Italy) to obtain the desired L and W (Figure 1A).

Slices were then sectioned from the blocks to the desired H of 1.3 mm using an Isomet diamond disc (Isomet 5000 precision saw, Buehler Ltd., Lake Bluff, IL, double-sided, 0.6 mm thickness, 45 mm diameter,) at 4,000 rotation per minute (rpm) under a water coolant. VS and E.max blocks were sliced directly at 1 mm thickness (H) using the same machine. The final thickness of the specimens was obtained by grinding the specimens with silicon carbide abrasive papers (380,600-grit) in a polishing machine under running water, and specimens that did not meet the desired dimensions were excluded. All procedures and measurements were performed by one investigator using a digital caliper. Crystallization for both E.max and VS and the sintering for PRT and PRTA were done adhering to the manufacturer’s recommendations. Final polishing was carried out for 20 seconds each under running water using 1600 Grit silicon carbide abrasive papers. The final thickness of the specimens was 1 mm (±0.05 mm). Before commencing with the test, all the specimens were cleaned for 10 minutes in an ultrasonic bath using distilled water and dried with compressed air (Figure 1B).

Measuring translucency

The translucency parameter (TP) was measured using a spectrophotometer (LabScan XE Spectrophotometer, Hunter Associates Laboratory Inc., Reston, VA) calibrated with white and black calibration tiles. This was considered as T1/baseline. International Commission on Illumination (CIE) L*a*b* values of each specimen were measured and light source illumination corresponding with average daylight (D65) was selected. TP was calculated by the color difference of the specimen measured against the white and black background. The L*, a*, b* values for the black and white backgrounds were (L= 0.01, a= -0.02. b= 0.01) and (L= 90.35, a= -1.31, b= -0.27), respectively. Measurements were recorded three times on each background using an aperture size of 5 mm and the mean CIE L*a*b* values were recorded. TP was calculated using the following TP formula [6]:

TP = [(L* _B _ - L*_W_)^2^+(a*_B _- a*_W_)^2^+(b*_B _- b*_W_)^2^]^1/2^

TP: translucency parameter (0-100), a higher TP value indicates more translucent material [6]. L* represents lightness, a* represents the red-green axis, b* represents the yellow-blue axis, B: color coordinates over the black background, W: over the white background.

Thermocycling

After the T1/baseline measurement was obtained, all the specimens were subjected to thermal aging using a thermocycling device (Thermocycler THE 1100 SD Mechatronik GmbH, Germany) at a temperature between 5°C and 55°C with 30s of dwell time and 10s of transfer time as proposed in ISO 11405 recommendations [12].

The following aging cycles were conducted:

(T2) for 10,000 thermocycles (1 year of clinical use), (T3) for 30,000 thermocycles (3 years of clinical use), and (T4) for 50,000 thermocycles (5 years of clinical use). Every 10,000 thermal cycles approximately correspond to one year of clinical function as Gale and Darvell postulated [13]. After each aging cycle (T2, T3, and T4), the specimens were removed from the distilled water, dried with paper towels, and then the L*a*b* coordinates were measured following the same methods as in T1. TP was then calculated at each interval using the TP formula.

Measuring color stability

Color stability was assessed by calculating Delta E (ΔE) using L*a*b* values against the black background only [14]. T1 black readings were used as the baseline parameter against which Delta E comparisons were made. The values of ΔE ≥3.3 were considered clinically unacceptable [14].

 The total color differences (ΔE) were calculated as follows:

ΔE = [(ΔL*)^2 ^+ (Δa*)^2 ^+ (Δb*)^2^]^1/2^

where L* is lightness, a* is green (-a), and red (+a) axis and b* is blue (-b) and yellow (+b) axis.

Scanning electron microscopy (SEM)

Random specimens from each group were coated with gold for SEM examination to inspect the grain size of the materials at baseline and after 50,000 thermocycles at 7000x.

Statistical analysis

Statistical analyses were done using SPSS software (Version 16.0, SPSS Inc., Chicago, IL). The levels, kurtosis, and skew for all variables used in the calculation were within normal limits, suggestive of a normally distributed sample. Therefore, parametric tests have been used in this study. One-way ANOVA was used to compare the four groups at each cycling interval, followed by Scheffe’s post-hoc test for intergroup comparisons (p<0.05). The overall ΔE and change in TP from interval to interval have been tested using multiple paired t-tests.

## Results

Translucency profile of the four materials

**Figure 2:**
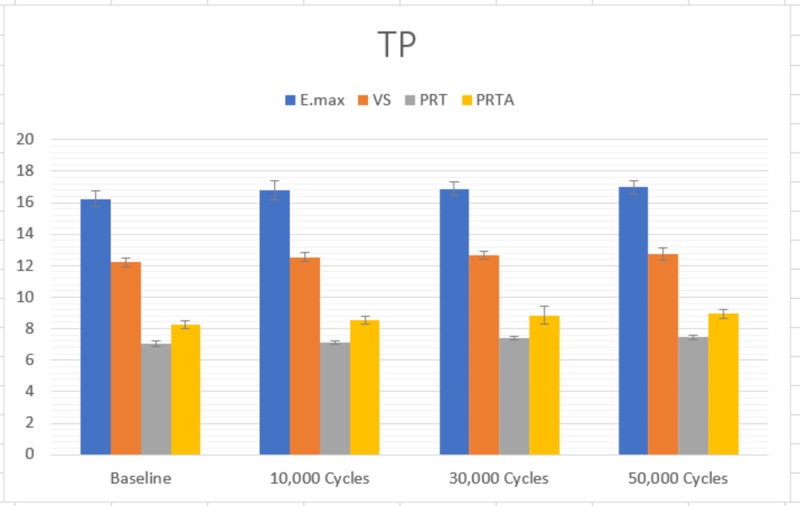
Translucency profile at each time period E.max: IPS e.max CAD®; VS: Vita Suprinity®; PRT: Prettau® 2 Dispersive®; PRTA: Prettau® 4 Anterior® Multi®

The one-way ANOVA showed that there were significant differences in the TP of the four materials at each time interval. The Scheffe’s post-hoc test showed that E.max had a significantly higher TP than VS, which had a significantly higher TP than PRTA (p<0.05). PRT had significantly lower TP than the other three materials (p<0.05). This was true for baseline, 10,000 thermocycles, 30,000 thermocycles, and 50,000 thermocycles (Figure 2). Each of the four materials studied showed a different pattern of change of translucency profile (Table 2). E.max showed a significant difference in TP from baseline to 10,000 cycles (p<0.001). Both VS and PRTA showed a significant change from baseline to 10,000 cycles (p<0.001), as well as from 10,000 to 30,000 cycles (p=0.001) and (p=0.002), respectively. The PRT group showed significant change in TP from 10,000 to 30,000 cycles (p<0.001) and from 30,000 to 50,000 cycles (p<0.05).

**Table 2:** Means and Std. deviations for the change in translucency profile for each time interval E.max: IPS e.max CAD®; VS: Vita Suprinity®; PRT: Prettau® 2 Dispersive®; PRTA: Prettau® 4 Anterior® Multi®; TP: translucency parameter (TP1: at baseline; TP2: after 10,000 cycles; TP3: after 30,000 cycles; TP4: after 50,000 cycles).* Differences significant at p<0.05

Material		Paired Differences		Sig.
		Mean	Std. Deviation	
E.max	TP1 - TP2	-.541	.340	.000*
	TP2 - TP3	-.078	.470	.323
	TP3 - TP4	-.104	.323	.061
VS	TP1 - TP2	-.319	.270	.000*
	TP2 - TP3	-.115	.182	.001*
	TP3 - TP4	-.057	.345	.323
PRT	TP1 - TP2	-.043	.198	.197
	TP2 - TP3	-.300	.117	.000*
	TP3 - TP4	-.033	.095	.041*
PRTA	TP1 - TP2	-.297	.195	.000*
	TP2 - TP3	-.318	.573	.002*
	TP3 - TP4	-.091	.577	.350

Color stability ΔE

**Figure 3:**
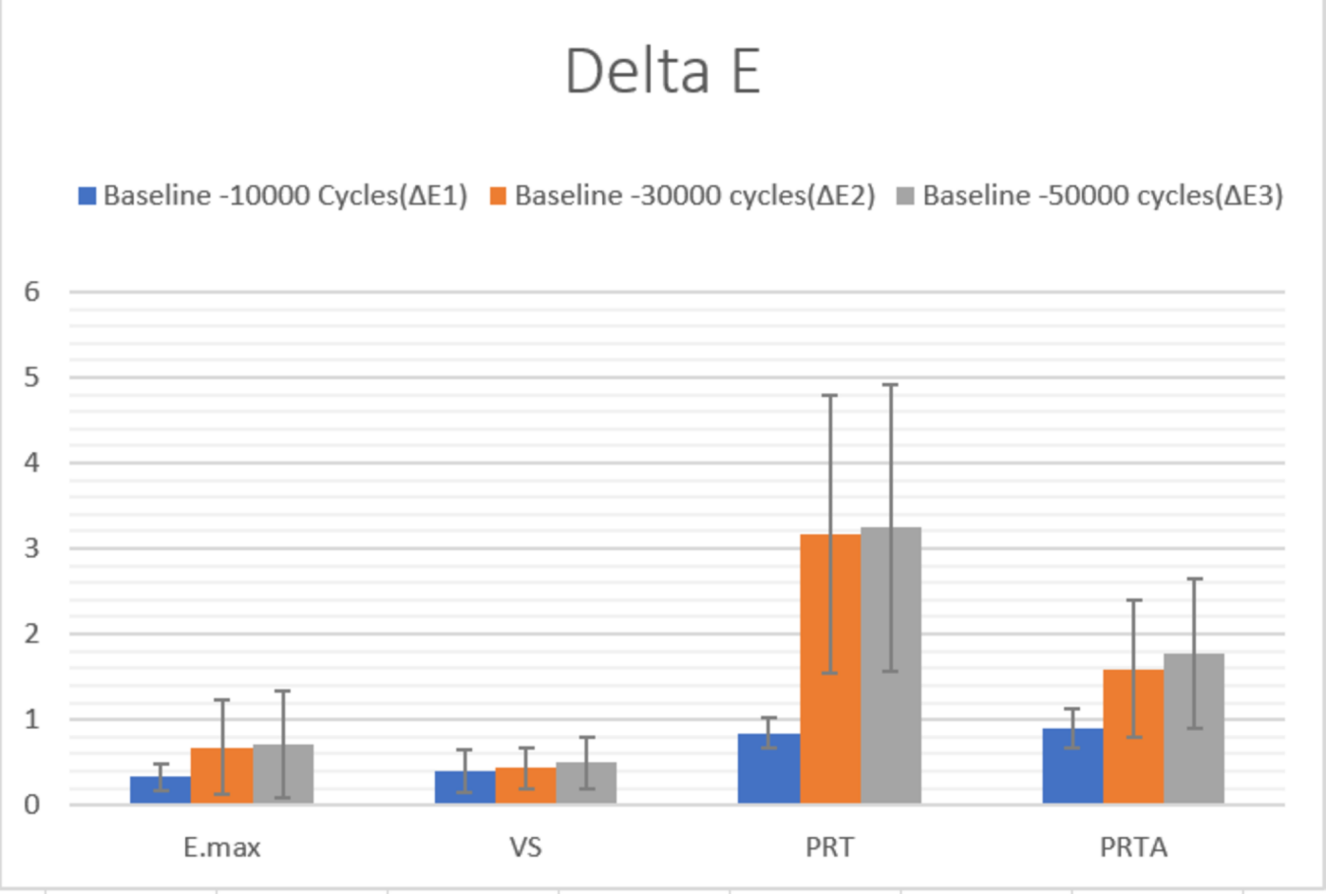
Delta E at each time interval

The one-way ANOVA showed a significant difference in color stability among the different materials at each of the time periods (p<0.001). From baseline to the end of 50,000 cycles, it was observed that there was no significant difference between the E.max and VS with ΔE1 (p=0.571), ΔE2 (p=0.753), ΔE3 (p=0.830) at which they had a significantly lower ΔE (p<0.05) than the PRT and PRTA. There was no significant difference in the ΔE1 between the PRT and PRTA (p=0.736). However, at ΔE2 and ΔE3, the PRTA had a significantly lower (p<0.05) delta E than the PRT (Figure 3).

When the difference in ΔE was compared from ΔE1 to ΔE3, it was observed that all four materials showed a significant difference in ΔE. E.max (p=0.001), PRT (p<0.001), and PRTA (p<0.001) showed a significant change from ΔE1 to ΔE2. In addition, PRT (p=0.013) and PRTA (p=0.012) showed a significant change from ΔE2 to ΔE3 (Table 3).

**Table 3:** Means and Std. deviations for the change in delta E per interval for each material E.max: IPS e.max CAD®; VS: Vita Suprinity®; PRT: Prettau® 2 Dispersive®; PRTA: Prettau® 4 Anterior® Multi®; ΔE, Color change (ΔE1: Baseline-10,000 cycles; ΔE2: Baseline-30,000 cycles; ΔE3: Baseline-50,000 cycles). ** Differences significant at p<0.05.

Material		Paired Differences		Sig.
		Mean	Std. Deviation	
E.max	ΔE1 - ΔE2	-.351	.592	.001**
	ΔE2 - ΔE3	-.040	.167	.160
	ΔE1 - ΔE3	-.391	.654	.001**
VS	ΔE1 - ΔE2	-.034	.318	.519
	ΔE2 - ΔE3	-.063	.335	.268
	ΔE1 - ΔE3	-.094	.163	.002**
PRT	ΔE1 - ΔE2	-2.322	1.687	.000**
	ΔE2 - ΔE3	-.078	.180	.013**
	ΔE1 - ΔE3	-2.400	1.741	.000**
PRTA	ΔE1 - ΔE2	-.696	.865	.000**
	ΔE2 - ΔE3	-.180	.407	.012**
	ΔE1 - ΔE3	-.877	.939	.000**

SEM micrographs

**Figure 4:**
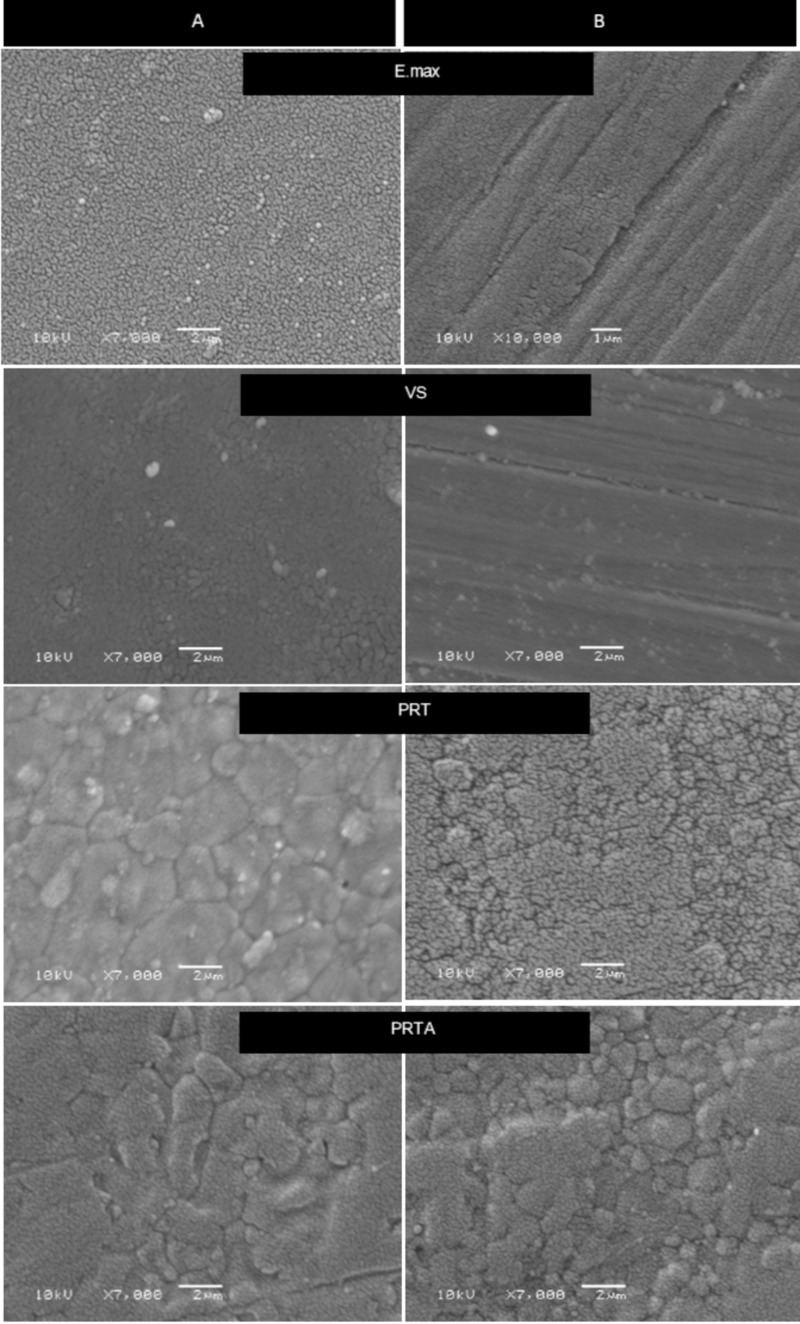
SEM photomicrographs (magniﬁcation ×7000) of the four groups A: indicates the surface at baseline; B: indicates the surface after 50,000 thermocycles. PRTA showed larger grains than PRT. SEM: scanning electron microscopy

Figure 4 shows SEM micrographs of the four ceramics before and after thermocycling. It presented regular surface morphology and polishing lines across the surfaces of both E.max and VS, which had smaller particles compared to PRTA and PTR. PRTA showed larger particle sizes as compared to PRT after thermocycling (Figure 4).

## Discussion

The goal of the present study was to examine the translucency of PRT, PRTA, and VS and to determine the effect of thermocycling on their translucency and color stability. Based on the results of this study, both null hypotheses were rejected.

The results showed that there were significant differences between all tested groups before and after the end of a total 50,000 aging cycles, with E.max having significantly highest TP values before and after aging, 16.2 and 16.9, respectively. This is in agreement with several studies [15-16]. Additionally, all the groups showed a significant increase in TP values by the end of the testing phase, although such increases did differ in pattern from one material to another.

The TP value of E.max CAD HT in this study was found to be slightly lower than in previous studies. Wang et al., reported TP of 19 for E-max CAD while Della Bona et al. reported 18.9 [6,17]. In contrast, Bagis and Turgut reported a TP value of about 14.49, which was lower than what was reported in the current study [18]. However, these values are closely related to human enamel and dentin TP. As mentioned by Yu B, at 1 mm thickness, the TP values of human enamel and dentin were 18.7 and 16.4, respectively [19]. Such differences could be related to several factors, one of which is the aperture size. Yu et al. used 3 mm while in the current study a 5 mm aperture was used for the measurement [19]. Other factors for difference in TP can be attributed to specimen thickness, sintering procedure, the different values of the white and black background used as well as the measuring devices [17,20-21].

The lower TP of the VS group compared to E.max is mostly related to its composition and the addition of 10% zirconia particles in its matrix. Notably, the values recorded in this study were higher than the ones reported by Alp and Subaşi (12.9 ±0.59 at 0.6 mm) [22].

Both zirconia groups presented the lowest values with TP at baseline of 8.2 for PRTA and 7.0 for PRT, with a statistically significant difference between them. These results concur with the manufacturer’s claim of enhancing the translucency of this new monolithic multilayer zirconia. However, they still showed less TP value than glass ceramics and this is related to the transmitted light through the zirconia, which was significantly lower than through the glass-based ceramics [23].

Although the composition of the zirconia used is unknown, based on the composition of the monochromatic form, it’s expected that the yttria content has been increased in PRTA, reaching up to 12 mol%, which produce more cubic form zirconia grains as compared with PRT where zirconia grains exists in tetragonal form. Increasing the cubic crystals improves zirconia’s translucency owing to optical anisotropy. SEM micrographs of PRTA showed a larger grain size as compared to PRT (Figure 4), resulting in decreased grain-boundary areas, which act as a scattering source [24]. This observation is in agreement with previous studies [17,25]. Kim and Kim, in their study, showed TP values for zirconia similar to the present study (Bruxzir was 10.29) [25]. However, these values were dissimilar to values reported by other authors [24,26]. Elsaka reported that the TP of Prettau Anterior as monolithic zirconia was 16.8, which was significantly lower than that for Ceramill Zolid FX (CZF) multilayer with 19.4 [24]. While, in another study, Sulaiman et al., reported the TP of 15.8 for Prettau Anterior as compared to 12.4 for Prettau Zirconia at 1 mm [26].

The mild increase in TP after aging in zirconia might be related to the grain size and the transformation to monoclinic form, which could be on the superficial layer only [27]. Similar findings have also been reported in previous studies [28-29]. Kim and Kim reported a significant increase in the translucency of both IPS e.max CAD and Katana as monolithic zirconia after autoclaving up to 10 hours [29]. Similarly, Sulaiman et al. reported the same increase in translucency after acidic aging treatment for 96 hours in a 37°C incubator for PSZ. This outcome is contrary to Abdelbary et al., who reported that the TP at 1 mm thicknesses was not significantly affected [30].

After aging the samples, they showed statistically significant differences in ΔE values between the four test materials, as well as within the same material at different time intervals. PRT showed significantly higher ΔE values, which could probably be attributed to the less yttria content compared to PRTA. However, it is worthy of note, that all the demonstrated ΔE values in all the groups were not clinically significant, at (ΔE < 3.3). This is in agreement with Alraheam et al. [20].

The number of cycles and aging methods reported in previous literature was inconsistent, making it difficult to co-relate the findings that have been previously reported. In general, different types of lithium disilicate and zirconia will have differences in their optical properties as documented by many authors and that these properties are often brand dependent [15,28]. Hence, no global statement can be made to generalize zirconia materials. However, these multicolored zirconia blocks transmit light differently within the block itself according to different layers. This can prove to be advantageous when compared to the monochromatic and non-shaded materials, thus enabling the planning of the position of the restoration within the block to achieve the desired shade and translucency.

Based on the results of this study, the authors suggest that the new multilayer zirconia can be a viable treatment alternative to achieve acceptable esthetic results in cases of minimal occlusal reduction, comparable with E.max and VS, which require a minimal thickness of 1.5 mm to 2.0 mm to avoid fracture. However, this advantage needs to be supported by further clinical studies to assure the claim. In addition, the results indicate that there might be slight color change and an increase in the translucency of the new monolithic materials that is expected to occur after five years of intraoral use, for which the authors recommend re-evaluating the esthetic match of restoration after five years of the cementation appointment.

One of the limitations of the current study was that only one brand of zirconia material was tested. Zirconia from multiple manufacturers has different formulations and chemical compositions, rendering different physical and optical properties between these materials. Thus, the results of this study cannot be generalized to other brands of Zirconia materials. The other is that although thermocycling was used to age the samples to simulate the intraoral condition, it does not replicate the intraoral environment holistically and what the restorative materials are exposed to other than the thermal factor.

## Conclusions

Within the limitations of the current study and based on the findings and behavior of materials with thermocycling, the following can be concluded: (1) E.max is significantly more translucent than all the other tested groups before and after thermocycling; (2) VS is significantly more translucent than PRTA followed by PRT, which had the least translucency; (3) The translucency of the tested materials is expected to increase intraorally with time; (4) Color changes after 50,000 cycles, though statistically significant, were within the clinically perceptible value and, therefore, clinically insignificant.
